# The effect of macronutrients on glycaemic control: a systematic review of dietary randomised controlled trials in overweight and obese adults with type 2 diabetes in which there was no difference in weight loss between treatment groups

**DOI:** 10.1017/S0007114515003475

**Published:** 2015-09-28

**Authors:** Amir Emadian, Rob C. Andrews, Clare Y. England, Victoria Wallace, Janice L. Thompson

**Affiliations:** 1 School of Sport, Exercise and Rehabilitation Sciences, University of Birmingham, Edgbaston, Birmingham B15 2TT, UK; 2 School of Clinical Sciences, University of Bristol, Bristol BS10 5NB, UK; 3 Centre for Exercise, Nutrition and Health Sciences, University of Bristol, Bristol BS8 1TZ, UK; 4 School of Psychology, University of Birmingham, Edgbaston, Birmingham B15 2TT, UK

**Keywords:** Type 2 diabetes, Systematic reviews, Diet, Weight loss

## Abstract

Weight loss is crucial for treating type 2 diabetes mellitus (T2DM). It remains unclear which dietary intervention is best for optimising glycaemic control, or whether weight loss itself is the main reason behind observed improvements. The objective of this study was to assess the effects of various dietary interventions on glycaemic control in overweight and obese adults with T2DM when controlling for weight loss between dietary interventions. A systematic review of randomised controlled trials (RCT) was conducted. Electronic searches of Medline, Embase, Cinahl and Web of Science databases were conducted. Inclusion criteria included RCT with minimum 6 months duration, with participants having BMI≥25·0 kg/m^2^, a diagnosis of T2DM using HbA1c, and no statistically significant difference in mean weight loss at the end point of intervention between dietary arms. Results showed that eleven studies met the inclusion criteria. Only four RCT indicated the benefit of a particular dietary intervention over another in improving HbA1c levels, including the Mediterranean, vegan and low glycaemic index (GI) diets. However the findings from one of the four studies showing a significant benefit are questionable because of failure to control for diabetes medications and poor adherence to the prescribed diets. In conclusion there is currently insufficient evidence to suggest that any particular diet is superior in treating overweight and obese patients with T2DM. Although the Mediterranean, vegan and low-GI diets appear to be promising, further research that controls for weight loss and the effects of diabetes medications in larger samples is needed.

Dietary intake is recognised as a major contributor to both the development and management of type 2 diabetes^(^ ^1^ ^)^. The current American Diabetes Association (ADA) recommendations for overweight and obese patients with type 2 diabetes mellitus (T2DM) include reducing energy intake while maintaining healthful eating patterns in order to promote weight loss^(^ ^2^ ^)^. Different diets have been studied to determine their impact on the management of T2DM. With regard to prevention, a recent meta-analysis of prospective cohort studies comprising 21 372 cases demonstrated that healthy diets (e.g. Mediterranean diet, dietary approaches to stop hypertension) were equally associated with a 20 % decreased risk of developing T2DM^(^ ^3^ ^)^. However, there remains no conclusive evidence as to which diet, if any, is the most effective in optimising glycaemic control in patients with T2DM^(^ ^4^ ^)^.

Two systematic reviews have examined the effects of different dietary interventions in managing T2DM. Ajala *et al.* ^(^ ^5^ ^)^ investigated the effects of low-carbohydrate, vegetarian, vegan, low glycaemic index (GI), high-fibre, Mediterranean and high-protein diets as compared with control diets (low fat, high GI, low protein, and diets described as following guidelines of the ADA or European Association for the Study of Diabetes). They concluded that the Mediterranean, low-carbohydrate, low-GI and low-protein diets resulted in greater improvements in HbA1c when compared with their respective controls, with the Mediterranean diet having the greatest effect. Meta-analyses also indicated that both the Mediterranean and low-carbohydrate diets produced the greatest weight loss (−1·84 and −0·69 kg, respectively).

Wheeler *et al*.^(^ ^6^ ^)^ conducted a systematic review that took a different approach. They examined the impact of macronutrients, food groups and eating patterns on diabetes management and risk for CVD. This was a follow-up to the literature review published by the ADA in 2001, and thus the authors only included studies published from 2001 to 2010. The authors concluded that many diets improved glycaemic control and cardiovascular risk factors; however, no one diet was identified as superior.

Both of these systematic reviews included studies in which the diets being examined resulted in greater weight loss than the respective ‘control diet’, making it difficult to determine whether the improvement in glycaemic control was due to weight loss or due to the composition of the diet. There is a need for a new systematic review to address this limitation. Thus, the aim of this systematic review was to analyse the results from only randomised controlled trials (RCT) where different dietary interventions were compared, and in which the total mean weight loss between groups was not statistically significantly different. If this analysis indicates significant improvements in glycaemic control, this would suggest that a particular diet may be more optimal for diabetes management.

## Methods

### Criteria for study consideration: types of studies and subjects

Only RCT with a minimum duration of 6 months and a measure of HbA1c were considered for this review, in order to examine long-term changes in HbA1c. The review set out to investigate the effects of dietary interventions in overweight and obese adults with T2DM; therefore, only studies in which subjects had a BMI of 25·0 kg/m^2^ or higher, along with a confirmed diagnosis of diabetes in line with the WHO diagnostic criteria^(^ ^7^ ^)^, were considered for inclusion. Studies needed to have at least two arms examining differences between dietary interventions. As the main aim of this study was to examine the impact of various diets on T2DM management independent of differential effects of weight loss, only trials in which there were no statistically significant differences in the mean weight lost between the arms were considered for inclusion. Studies including pharmacological or physical activity interventions were excluded. Only interventions using a whole-diet approach were of interest, and hence trials involving individual foods, functional foods or individual supplements were excluded.

### Outcome measures

The main outcome of interest for this review was the mean difference in HbA1c between dietary arms at the end point of intervention.

### Search strategy

Electronic searches were conducted in Medline, Embase, Cinahl and Web of Science databases including all studies published as of 29 June 2015. References of included studies along with published reviews were hand searched for additional studies. Individual search strategies were developed according to the specifications of the different databases. A combination of exploded medical subject headings (MeSH) and free text searching was used as part of the search strategies. MeSH headings that were used included ‘Type 2 diabetes’, ‘NIDDM’, ‘Haemoglobin A, Glycosylated’, ‘Diet’, ‘Dietary proteins’, ‘Dietary fats’, ‘Dietary carbohydrates’, ‘Glycaemic index’, Glycaemic load’ and their variants. The search was limited to studies written in the English language (see online Supplementary Appendix S1).

V. W. who was our research librarian was instrumental in working with the lead author (A. E.) to develop and finalise the search strategy for the four databases. A. E. screened all titles and abstracts and initially assessed studies for inclusion. Where it was unclear whether a study met the inclusion criteria, a second author (J. L. T.) screened the reports.

### Study quality assessment and data extraction

The lead author (A. E.) rated the quality of the RCT identified by the searches using the Joanna Briggs Institute^(^ ^8^ ^)^ critical appraisal tool to ensure trials were of a sufficient quality (see online Supplementary Appendix S2). A second independent reviewer rated the quality of a sub-sample of twenty relevant articles. Data extraction was then conducted by A. E. and an independent reviewer on the final eleven articles that met all inclusion criteria, using a custom-designed data extraction sheet.

As the published studies lacked a common control diet for comparison, it was not possible to conduct a meta-analysis of the results from the included studies. Thus, the results of a qualitative synthesis are reported here.

## Results

### Study selection

**Fig. 1:**
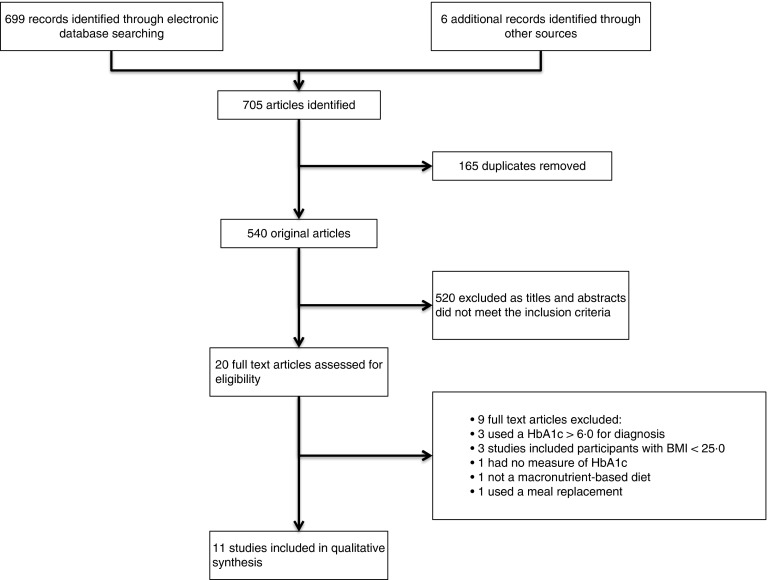
Flow diagram showing the number of studies screened, assessed for eligibility, and included in the review.

Through initial electronic database searching and hand searching, 705 studies were identified (Fig. 1). After removal of duplicates, this was reduced to 525 studies. The initial stage of assessing studies focused on excluding studies based on information present in the titles and abstracts, which resulted in the elimination of 540 studies. A total of twenty remaining studies were then accessed in full text form to further assess eligibility. Of these twenty studies, nine were excluded as they failed to meet one or more of the inclusion criteria (Fig. 1). The remaining eleven studies met all inclusion criteria and after critical appraisal were deemed to meet the quality requirements to be included in the qualitative synthesis.

### Study and subject characteristics

The eleven studies included in this review are summarised in Table 1. The duration of the interventions ranged from 40 weeks^(^ ^9^ ^)^ to 4 years^(^ ^10^ ^)^. The trials varied in size, with the smallest study including forty^(^ ^11^ ^)^ participants and the largest study including 259^(^ ^12^ ^)^ participants. The pooled sample size for all studies was *n* 1266.

**Table 1:** Table summarising the results of changes in HbA1c from the eleven dietary interventions included in the systematic review (Mean values and standard deviations for mean weight loss and mean reduction HbA1c)

		HbA1c (%) at baseline									
References	Participants	Mean	sd	Intervention (*n* per arm)	Composition of prescribed diets	Mean weight loss (kg)	Mean decrease in HbA1c (%)	Duration	Attrition rate	Medication	Conclusion
Guldbrand *et al*.^(^ ^18^ ^)^	61 overweight and obese adults with type 2 diabetes	7·35		LFD (31) *v*. LCD (30)	LFD: 55–60 % CHO, 10–15 % protein, 30 % fat LCD: 20 % CHO, 30 % protein, 50 % fat	2·97 *v*. 2·34 (*P*=0·33)	−0·2 *v*. 0: no significant difference between groups (*P*=0·76)	2 years	9·7 % in LFD group and 13·3 % in LCD	Authors reported that at 6 months there was a statistically significant difference in mean insulin dose in favour of the LCD (*P*=0·046)	No significant difference between dietary interventions in improving HbA1c
Brehm *et al.* ^(^ ^19^ ^)^	124 overweight and obese adults with type 2 diabetes	7·3		High-MUFA diet (43) *v*. HC diet (52)	MUFA: 45 % CHO, 15 % protein, 40 % fat (20 % MUFA) CHO: 60 % CHO, 15 % protein, 25 % fat	4·0 (sd 0·8) *v*. 3·8 (sd 0·6) (*P*=0·867)	0 *v*. *–*0·1: authors stated no significant difference between groups (no *P* value reported)	12 months	31 % for the MUFA group and 16 % for the HC group	Authors reported a lack of available information about participant’s drug usage. Only information on 32 participants’ drug use was available, which showed no systematic differences between diet groups. Therefore no adjustments were made for glucose-lowering medication	No significant difference between dietary interventions in improving HbA1c
Fabricatore *et al*.^(^ ^9^ ^)^	79 obese adults with type 2 diabetes	6·8		Low fat (39) *v*. low GL (40)	Low fat: <30 % fat Low GL: 3 or less servings of moderate-GL and 1 or less serving of high-GL foods/d	4·5 (sd 0·34) *v*. 6·4 (sd 0·52) (*P*=0·28)	0·1 (sd 0·0012) *v*. 0·8 (sd 0·0104): significant difference between groups (*P*=0·01)	40 weeks	36·7 %	Authors stated that changes in HbA1c were adjusted for medication use. Percentage of participants who increased, decreased or did not change their diabetic medication regime did not differ between the groups at week 20 (*P*=0·51) or at week 40 (*P*=0·70)	Low GL appears to be more effective in reducing HbA1c compared with a the LFD
Elhayany *et al*.^(^ ^12^ ^)^	259 overweight and obese adults with type 2 diabetes	8·3		LCMD (61) *v*. TM diet (63) *v*. ADA diet (55)	LCM: 35 % low-GI CHO, 15–20 % protein, 45 % fat rich in MUFA TM: 50–55 % low-GI CHO, 15–20 % protein, 30 % fat rich in MUFA ADA: 50–55 % CHO, 15–20 % protein, 30 % fat	10·1 *v*. 7·4 *v*. 7·7 authors stated no significant difference between groups (no *P* value reported)	2·0 *v*. 1·8 *v*. 1·6 significant difference between diets (*P*=0·021), LCM different than ADA, TMD different than ADA	12 months	30·9 %	Authors do not mention baseline medication characteristics or any changes in glucose-lowering medication use during the course of the intervention	LCM diet appears to be more effective in reducing HbA1c compared with a TM and ADA diets
Barnard *et al*.^(^ ^13^ ^)^	99 obese adults with type 2 diabetes	7·99		Low-fat vegan diet (49) *v*. ADA diet (50)	Low-fat vegan diet: 75 % CHO, 15 % protein, 10 % fat ADA: 60–70 % CHO, 15–20 % protein and MUFA	4·4 (sd 0·9) *v*. 3·0 (sd 0·8) (*P*=0·25)	0·34(sd 0·19) *v*. 0·14 (sd 0·17) no significant difference between groups (*P*=0·43) 0·4 % *v*. 0·01 % (*P*=0·03) when adjusted for medication	74 weeks	18·4 % for the vegan group 14 % for the ADA group	Net 74-week dosages were reduced in 35 % participants in vegan group and 20 % of those in the ADA group, and were increased in 14 % of vegan group and 24 % of conventional group	Once data are adjusted for medication use, there appears to be a significant benefit in the low-fat vegan diet in decreasing HbA1c compared with the ADA diet
Esposito *et al*.^(^ ^10^ ^)^	215 overweight adults with type 2 diabetes	7·73		LCMD (107) *v*. LFD (108)	LCMD: 50 % CHO, 20 % protein, no <30 % fat LFD: no >30 % fat with no >10 % SFA	3·8 (sd 2) *v*. 3·2 (sd 1·9) authors stated no significant difference between groups (no *P* value reported)	0·9 (sd 0·6) *v*. 0·5 (sd 0·4) authors stated significant differences between groups (no *P* value reported)	4 years	9·3 %	After 4 years 44 % of participants in the LCMD and 70 % of those in the LFD group required treatment (absolute difference, −26·0 percentage points (95 % CI 0·51, 0·86), hazard ratio adjusted for weight change, 0·70 (95 % CI 0·59, 0·90); *P*<0·001).	LCMD appears to be more effective in reducing HbA1c compared with a LFD with less need for glucose-lowering medication
Pedersen *et al*.^(^ ^14^ ^)^	76 overweight adults with type 2 diabetes	7·3		HPD (21) *v*. standard protein diet (24)	HPD: 40 % CHO, 30 % protein, 30 % fat SPD: 50 % CHO, 20 % protein, 30 % fat	9·7 (sd 2·9) *v*. 6·6 (sd 1·4) (*P*=0·32)	0·4 *v*. 0·3 no significant difference between groups (*P*=0·29)	12 months	40·8%	Did not account for changes in medication, although authors stated that 4 volunteers managed their diabetes with diet alone, and all others treated with oral medication and/or insulin	No significant difference between dietary interventions in improving HbA1c
Iqbal *et al*.^(^ ^17^ ^)^	144 obese adults with type 2 diabetes	7·75		LCD (40) *v*. LFD (28)	Low CHO: <30 g/d CHO Low fat: ≤30 % of energy for fat with <7 % of energy from SFA	1·5 *v*. 0·2 (*P*=0·147)	0·1 *v*. 0·2 no significant difference between groups (no *P* value reported)	24 months	60 % in the low-CHO group and 46 % in the low-fat group	Authors stated that many participants were unable to provide information regarding changes to medication or dosages and therefore the effects of glucose-lowering medication was not adjusted for	No significant difference between dietary interventions in improving HbA1c
Ma *et al*.^(^ ^11^ ^)^	40 overweight and obese adults with type 2 diabetes	8·42		ADA diet (21) *v*. low-GI diet (19)	ADA: 60–70 % CHO, 15–20 % protein and 30 % fats Low GI: participants given goals to reduce daily dietary GI score to 55	0·80 *v*. 1·32 (*P*=0·89)	0·43 *v*. 0·35 no significant difference between groups (*P*=0·88)	12 months	10 % for the ADA group and 10 % for the low-GI group	Participants in the low-GI group were less likely to add or increase dosage of glucose-lowering medications (OR 0·26; *P*=0·01)	No significant difference between dietary interventions in improving HbA1c
Milne *et al*.^(^ ^15^ ^)^	70 overweight and obese adults with type 2 diabetes	10·0	0·35	Weight management diet (21) *v*. HC/fibre diet (21) *v*. modified lipid diet (22)	Weight management diet: restrict extrinsic simple sugars and energy dense foods, no advice for macronutrient contribution HC/fibre: 55 % CHO, 15 % protein, 30 % fat, 30 g or more dietary fibre/d Modified lipid diet: 45 % CHO, 19 % protein, 36 % fat	−1·5 *v*. 1·0 *v*. 0·1 authors stated no significant difference between groups (no *P* value reported)	0·1 *v*. 0·1 *v*. 0·2 authors stated no significant difference between groups (no *P* value reported)	18 months	8·6 %	Authors state that 52·3 % of the weight management group, 57·1 % of the HC/fibre group and 50 % of the modified lipid diet were on glucose-lowering medication at baseline. However there is no mention of medication adjustments being made throughout the study	No significant difference between dietary interventions in improving HbA1c
Larsen *et al*.^(^ ^16^ ^)^	99 overweight and obese adults with type 2 diabetes	7·84		HPD (53) *v*. HC diet (46)	HP: 40 % CHO, 30 % protein, 30 % fat HC: 55 % CHO, 15 % protein, 30 % fat	2·23 *v*. 2·17 (*P*=0·78)	0·23 *v*. 0·28 no significant difference between groups (*P*=0·44)	12 months	9·2 % HP group *v*. 4·2 % HC	Authors reported a significant reduction in the requirement for glucose-lowering medication in the HP group compared with the HC group at 3 months (*P*=0·03) although the difference was no longer significant at 12 months (*P*=0·05). However authors stated that that there were not significant differences in the decrease in HbA1c between groups when values were adjusted for changes in medication	No significant difference between dietary interventions in improving HbA1c

LFD, low-fat diet; LCD, low carbohydrate diet; CHO, carbohydrate; HC, high carbohydrate; low GL, low glycaemic load; LCMD, low carbohydrate Mediterranean diet; TM, traditional Mediterranean; ADA, American Diabetes Association; LCM, low carbohydrate Mediterranean; GI, glycaemic index; TMD, traditional Mediterranean diet; HPD, high-protein diet; SPD, standard protein diet; HP, high protein.

### Interventions: general overview

A wide range of dietary interventions were examined, including low-fat vegan, ADA, low GI, high-protein diet, standard protein diet, low-fat diet, low carbohydrate, low glycaemic load (GL), low carbohydrate Mediterranean (LCM), traditional Mediterranean (TM), high carbohydrate/fibre and a modified lipid diet. Of the eleven studies included, two compared three different dietary interventions, whereas the other nine studies compared two dietary interventions. In total there were twenty-four individual comparators.

### Interventions showing a positive effect

From the eleven studies, nine demonstrated a positive effect of dietary intervention on improving HbA1c values at the end point of intervention^(^ ^9^ ^–^ ^17^ ^)^. However, five of these studies did not report statistically significant differences between dietary arms in the reductions in HbA1c values^(^ ^11^ ^,^ ^14^ ^–^ ^17^ ^)^, and hence do not appear to support the use of one dietary intervention over another, as comparators had similar positive effects on glycaemic control.

### Interventions showing no effect

Out of the eleven studies, two reported that the prescribed dietary interventions failed to decrease HbA1c levels^(^ ^18^ ^,^ ^19^ ^)^. Guldbrand *et al.* ^(^ ^18^ ^)^ compared a low-carbohydrate diet with a low-fat diet, and despite both groups experiencing significant weight loss there were no significant improvements in HbA1c at the end point of either dietary intervention. However, the authors stated that at 6 months into the intervention there was a statistically significant difference in mean insulin dose in favour of the low-carbohydrate diet (*P*=0·046). Brehm *et al.* ^(^ ^19^ ^)^ compared a predominantly MUFA diet with a high-carbohydrate diet, and again despite reductions in body weight over 12 months of 4·0 (sd 0·8) *v*. 3·8 (sd 0·6) kg, respectively, the interventions failed to be effective in improving glycaemic control, with non-significant mean changes in HbA1c levels for both groups. It is important to note that authors reported a lack of information about changes that were made to the type and dosage of glucose-lowering medication. Therefore, it appears that no adjustments were made to account for the effects of medication on glycaemic control. This lack of ability to take into consideration the effect of medication on glycaemic control is a potential limitation.

### Interventions showing significant differences between dietary groups

Only four studies reported a significant difference in HbA1c between different dietary interventions despite a non-significant difference in weight loss (Table 1)^(^ ^9^ ^,^ ^10^ ^,^ ^12^ ^,^ ^13^ ^)^.

Fabricatore *et al.* ^(^ ^9^ ^)^ compared a low-fat diet with a low-GL diet, with the subjects in the low-GL group experiencing a significantly greater reduction in HbA1c compared with those in the low-fat diet group: 0·8 (sd 0·0104) *v*. 0·1 (sd 0·0012) %, respectively (*P*=0·01). Authors reported that the values presented were adjusted to account for changes in glucose-lowering medication, and that the percentage of participants who increased, decreased or did not change their medication protocol was not statistically different between groups at week 20 (*P*=0·51) or at week 40 (*P*=0·70). Therefore, this study appears to demonstrate a benefit of a low-GL diet over a low-fat diet in improving HbA1C levels.

Elhayany *et al.* ^(^ ^12^ ^)^ conducted a three-arm intervention comparing a LCM diet, a TM diet and the 2003 ADA diet. All three interventions were successful in reducing weight and improving HbA1c levels. Subjects in the LCM diet experienced the greatest reduction in HbA1c: 2·0 % compared with 1·8 % in the TM group and 1·6 % in the ADA group (*P*=0·021). However, it is important to view these results with caution, as authors do not report baseline medication characteristics of the participants or any changes in glucose-lowering medication throughout the course of the intervention. Therefore, values have not been adjusted for medication, and the lack of information available regarding type and dosage of glucose-lowering medication makes it impossible to confirm that it was the LCM diet itself that was more effective in reducing HbA1c, or whether the changes observed may have been a result of differences in medication use and dosage between the three intervention groups.

Barnard *et al.* ^(^ ^13^ ^)^ compared a low-fat vegan diet with an ADA diet, with results showing a greater mean reduction in HbA1c for patients on the low-fat vegan diet. Authors reported a mean decrease of 0·4 % for the low-fat vegan group and 0·1 % decrease for the ADA group once adjustments were made for changes in medication.

Esposito *et al.* ^(^ ^10^ ^)^ compared an LCM diet with a low-fat diet. The LCM diet led to a significantly greater reduction in Hba1c, with a mean decrease of 0·9 % compared with the 0·5 % achieved in the low-fat diet group. This study appears to show a benefit of using an LCM diet over a low-fat diet in reducing Hba1C levels beyond the effects of weight loss. Two of the strengths of this study are that all participants were newly diagnosed with T2DM and were not taking any form of glucose-lowering medication. The primary outcome of the study was commencement of medication, which itself followed a strict protocol. As shown in Table 1, the LCM diet resulted in a significantly lower HbA1c value with less need for glucose-lowering medication when compared with the low-fat diet (LFD). However, because of the nature of the study design, physicians were not blinded to the intervention groups in order to administer medication, which is a limitation.

### Limitations in adherence to prescribed diets

One issue common to most studies was the lack of compliance to the prescribed dietary intervention. As shown in Table 2, apart from Pedersen *et al.* ^(^ ^14^ ^)^ who used the 24-h urea excretion method for assessing adherence to prescribed protein intakes, the remaining ten studies relied on self-report dietary intake data. Differences in prescribed *v*. reported diets are apparent when comparisons are made with the macronutrient targets set at baseline to those that were reported at the end point of intervention (Table 2). Pedersen *et al.* ^(^ ^14^ ^)^ reported that adjusted urea excretion was significantly different between groups (519 (sd 39) for the high-protein diet and 456 (sd 25) for the standard protein diet group; *P*=0·04), indicating compliance to the protein prescription. In contrast, Iqbal *et al.* ^(^ ^17^ ^)^ reported no significant difference in macronutrient intake between groups at any point during the intervention. In this study the subjects in the low-carbohydrate group were prescribed a diet with <30 g of carbohydrates/d; however, data from 3-d food diaries revealed a mean carbohydrate intake of 192·8 g/d. Similarly, Barnard *et al.* ^(^ ^13^ ^)^ reported that, at the end point of intervention, dietary adherence was met by only 51 % of those in the low-fat vegan group and by 48 % of those in the ADA group.

**Table 2:** Table summarising the changes in dietary intake at baseline and at end point of intervention

References	Intervention (*n* per arm)	Dietary assessment tool used	Composition of prescribed diet	Composition of diet consumed*	Comments
Guldbrand *et al*.^(^ ^18^ ^)^	LFD (31) *v*. LCD (30)	3-d food record	LFD: 55–60 % CHO, 10–15 % protein, 30 % fat	LFD: 47 % CHO, 20 % protein, 31 % fat	There was a significant between group differences for the percentage of energy from CHO, fat (*P*<0·001) and for protein (*P*=0·009)
			LCD: 20 % CHO, 30 % protein, 50 % fat	LCD: 31 % CHO, 24 % protein, 44 % fat	
Brehm *et al*.^(^ ^19^ ^)^	High-MUFA diet (43) *v*. high-CHO (CHO) diet (52)	3-d food record	MUFA: 45 % CHO, 15 % protein, 40 % fat (20 % MUFA)	MUFA: 46 % CHO, 16 % protein, 38 % fat (20 % MUFA)	The high-MUFA-diet group consumed significantly more total fat, PUFA and MUFA than the high-CHO group (*P<*0·001)
			CHO: 60 % CHO, 15 % protein, 25 % fat	CHO: 54 % CHO, 18 % protein, 28 % fat	
Fabricatore *et al*.^(^ ^9^ ^)^	Low fat (39) *v*. low GL (40)	3-d food record	Low fat: <30 % fat	Low fat: 32·9 % fat GL 121·3	Reductions in energy from fat were significantly greater among those in the low-fat group at week 40 (*P*≤0·01)
			Low GL: 3 or less servings of moderate-GL and 1 or less serving of high-GL foods/d	Low GL: 39·8 % fat GL 88·6	Low-GL group had significantly greater reductions in energy from CHO (*P*≤0·01) and significantly greater reductions in dietary GI (*P*≤0·003) and GL (*P*≤0·03)
					Changes on other measured dietary variable were not significantly different between groups
Elhayany *et al*.^(^ ^12^ ^)^	LCM (61) *v*. TM (63) *v*. ADA diet (55)	FFQ and 24-h recall	LCM: 35 % low-GI CHO, 15–20 % protein, 45 % fat rich in MUFA	No breakdown of the actual macronutrient content which was consumed during the intervention	Statistically significant trend in percentage of energy from PUFA intake, highest 12·9 % for LCM, to 11·5 % in TM, and lowest in ADA 11·2 % (*P*=0·002)
			TM: 50–55 % low-GI CHO, 15–20 % protein, 30 % fat rich in MUFA		Same significant trend observed for MUFA fat intake (14·6, 12·8, and 12·6 % for LCM, TM and ADA, respectively, *P*<0·001).
			ADA: 50–55 % CHO, 15–20 % protein, 30 % fat		Opposite trend seen for % of energy from CHO, highest for ADA 45·4 % then 45·2 % for TM and lowest for the LCM with 41·9 % (*P*=0·011)
Barnard *et al*.^(^ ^13^ ^)^	Low-fat vegan diet (49) *v*. ADA diet (50)	3-d food record	Low-fat vegan diet: 75 % CHO, 15 % protein, 10 % fat	Low-fat vegan diet: 66·3 % CHO, 14·8 % protein, 22·3 % fat	At the end point of intervention, dietary adherence was met by 51 % of participants in the vegan group and 48 % of those in the ADA group
			ADA: 60–70 % CHO, 15–20 % protein and 30 % fats	ADA: 46·5 % CHO, 21·14 % protein and 33·7 %	
Esposito *et al*.^(^ ^10^ ^)^	LCMD (107) *v*. LFD (108)	Food diary records	LCMD: 50 % CHO, 20 % protein, no <30 % fat	LCMD: 44·2 % CHO, 18 % protein, 39·1 % fat, 10 % SFA, 17·6 % MUFA	Between group differences in % CHO and MUFA significantly different throughout the trial
			LFD: no >30 % fat with no >10 % SFA	LFD: 51·8 % CHO, 17·9 % protein, 29·4 % fat, 9·4 % SFA, 12·4 % MUFA	
Pedersen *et al*.^(^ ^14^ ^)^	HPD (21) *v*. SPD (24)	FFQ and 24 h urea excretion	HPD: 40 % CHO, 30 % protein, 30 % fat	HPD: 39·2 % CHO, 26 % protein, 34·8 % fat	At 12 months adjusted urea excretion was significantly different between groups (519 (sd 39) for HPD and 456 (sd 25) for the SPD group, *P*=0·04) indicating compliance to protein prescription
			SPD: 50 % CHO, 20 % protein, 30 % fat	SPD: 44·8 % CHO, 21·1 % protein, 34·0 % fat	
Iqbal *et al*.^(^ ^17^ ^)^	LCD (40) *v*. LFD (28)	3-d food record	LCD: *<*30 g/d CHO	LCD: 192·8 g/d CHO	Authors concluded that macronutrient intake was not significantly different between groups at any point. Authors concluded that both groups failed to achieve dietary targets
			Low fat: ≤30 % of energy for fat with <7 % of energy from SFA	Low fat: 33·6 % of energy from fat	
Ma *et al*.^(^ ^11^ ^)^	ADA diet (21) *v*. low-GI diet (19)	7-d dietary recall	ADA: 60–70 % CHO, 15–20 % protein and 30 % fats	ADA: 38 % CHO, 80 GI, 20 % protein, 43 % fat	Differences in dietary GI did not reach significance until 12 months (*P*=0·07), however GL was significantly lower in the low-GI diet at 6 months (97 *v*. 141; *P*=0·02)
			Low GI: participants given goals to reduce daily dietary GI score to 55	Low GI: daily GI score of 76 37 % CHO, 76 GI, 20 % protein, 43 % fat	
Milne *et al*.^(^ ^15^ ^)^	Weight management diet (21) *v*. high-CHO/fibre diet (21) *v*. modified lipid diet (22)	24-h recall	Weight management diet: restrict extrinsic simple sugars and energy dense foods, no advice for macronutrient contribution	Weight management diet: 47·6 % CHO, 18·8 %, 33·6 %, 17·3 g dietary fibre/d	Authors concluded that almost none of the participants succeeded in achieving currently recommended intakes of either CHO or unsaturated fat
			High CHO/fibre: 55 % CHO, 15 % protein, 30 % fat, 30 g or more dietary fibre/d	High CHO/fibre: 46·6 % CHO, 21·0 % protein, 32·4 % fat, 21·1 dietary fibre/d	
			Modified lipid diet: 45 % CHO, 19 % protein, 36 % fat	Modified lipid diet: 46·4 % CHO, 19·7 % protein, 33·9 % fat	
Larsen *et al*.^(^ ^16^ ^)^	HPD (53) *v*. high-CHO diet (46)	3-d food record	HPD: 40 % CHO, 30 % protein, 30 % fat	HPD: 41·8 % CHO, 26·5 % protein, 30·7 % fat	Significant differences between groups in the quantities of CHO and protein consumed
			High CHO: 55 % CHO, 15 % protein, 30 % fat	High CHO: 48·2 % CHO, 18·9 % protein, 32·0 % fat	

LFD, low-fat diet; LCD, low carbohydrate diet; CHO, carbohydrate; low GL, low glycaemic load; GI, glycaemic index; LCM, low carbohydrate Mediterranean; TM, traditional Mediterranean; ADA, American Diabetes Association; LCMD, low carbohydrate Mediterranean diet; HPD, high-protein diet; SPD, standard protein diet.

* Values are from reported dietary intakes at the end point of intervention.

## Discussion

The results of this systematic review indicate that only four out of the eleven trials demonstrated a benefit of one particular dietary intervention over another. These diets were low GL, LCM and low-fat vegan. Therefore, it appears that these diets may have a beneficial effect on HbA1c independent of weight loss. However, there are two major limitations within most of these studies that could have substantially affected the reported results: lack of reporting and controlling for medication use and change, and poor compliance to the dietary intervention being studied.

Elhayany *et al.* ^(^ ^12^ ^)^ demonstrated that the low carbohydrate Mediterranean diet (LCMD) was more effective than the TMD and the ADA diet in reducing HbA1c. However, this study lacked any control over the effects of glucose-lowering medication, with no information available about baseline medication or any changes to medication occurring during the trial. Therefore, we cannot be certain whether the effects on the outcome measures were due to the dietary intervention or due to effects of glucose-lowering medication.

The three interventions that show promise appear to be those of Fabricatore *et al.* ^(^ ^9^ ^)^, who demonstrated the benefit of a low-GL diet over a low-fat diet, Barnard *et al.* ^(^ ^13^ ^)^ who demonstrated a potential benefit of a low-fat vegan diet compared with the ADA diet, and Esposito *et al.* ^(^ ^10^ ^)^ who showed a benefit of using an LCM diet over a low-fat diet. In contrast to the study by Elhayany *et al.* ^(^ ^12^ ^)^ these three studies reported how changes in glucose-lowering medication were managed and accounted for throughout the interventions. Furthermore, both Barnard *et al.* ^(^ ^13^ ^)^ and Esposito *et al.* ^(^ ^10^ ^)^ reported that HbA1c values were significantly reduced, with less need for glucose-lowering medication in the low-fat vegan and LCM dietary groups. Fabricatore *et al*.^(^ ^9^ ^)^ demonstrated a benefit of using a low-GL diet compared with a low-fat diet; however, a limitation of this intervention was the high attrition rate of 36·7 %.

Although the mechanisms leading to enhanced glycaemic control in these studies were not examined, existing research may help explain their findings. One potential mechanism for the effectiveness of a low-fat vegan diet is its high dietary fibre content. By the end of the 74-week intervention, subjects in the low-fat vegan group were consuming a significantly greater amount of dietary fibre than were those in the ADA group (21·7 (sd 1·2) *v*. 13·4 (sd 0·8) g/4184 kJ (1000 kcal)). Both Post *et al.* ^(^ ^20^ ^)^ and Silva *et al.* ^(^ ^21^ ^)^ conducted meta-analyses demonstrating the benefits of increasing fibre intakes and improved glycaemic control in patients with T2DM. Although these meta-analyses did not control for energy consumption, they do highlight the importance of dietary fibre in diabetes management. This is of importance when considering the effects of dietary approaches such as low-fat vegan or Mediterranean diets, as dietary fibre intakes tend to increase when consuming these diets, and as such any observed benefits on glycaemic control may potentially be due to increased fibre consumption.

A component of the Mediterranean diet that has been highlighted as a possible mechanism for its benefit in optimising glycaemic control is the increased intake of MUFA. Esposito *et al.* ^(^ ^10^ ^)^ reported a significant increase in the percentage of energy from MUFA in participants consuming the LCMD compared with the LFD. Paniagua *et al.* ^(^ ^22^ ^)^ conducted a prospective crossover study on eleven insulin-resistant subjects, each spending 28 d consuming a diet high in SFA, a diet high in MUFA and a diet high in carbohydrates. The MUFA-rich diet improved insulin sensitivity, and lowered insulin resistance (homoeostasis model assessment-insulin resistance) to a greater extent compared with the high-SFA and the high-carbohydrate diets (2·32 (sd 0·3), 2·74 (sd 0·4), 2·52 (sd 0·4), respectively, *P*<0·01). The high-MUFA diet also increased glucagon-like peptide-1 (GLP-1) more than did the carbohydrate-rich diet. The diets were designed to ensure weight maintenance, with no changes in patients’ body weights reported. Therefore, this study demonstrated a potential effect of MUFA in improving insulin sensitivity, possibly through increased GLP-1 levels, independent of weight change.

The current systematic review does not fully support the findings of the previous systematic review conducted by Ajala *et al.* ^(^ ^5^ ^)^, as our findings do not support any benefit of consuming low-carbohydrate or high-protein diets over another dietary intervention. Similar to the results of Ajala *et al*.^(^ ^5^ ^)^, our findings suggest a potential benefit of a Mediterranean-style diet. Three trials were included in their analysis, two of which were included in the current systematic review (Elhayany *et al.* ^(^ ^12^ ^)^, Esposito *et al.* ^(^ ^10^ ^)^), with the third (Toobert *et al* ^(^ ^23^ ^)^) not meeting the inclusion criteria of the current systematic review as weight loss between groups was statistically significantly different. In addition, it is important to note that in the study by Toobert *et al.* ^(^ ^23^ ^)^ participants randomised to the Mediterranean Lifestyle Program were not only given dietary advice to follow a Mediterranean diet but were also given stress management classes, with exercise prescriptions involving both aerobic and strength-training activity. Therefore, the beneficial effects on HbA1c could have been due to many components of the intervention and not just dietary change. Therefore, considering this study, along with those of Esposito *et al.* ^(^ ^10^ ^)^ and Elhayany *et al.* ^(^ ^12^ ^)^ (who did not take into account changes in medication), makes it difficult to assess the potential use of the meta-analysis conducted by Ajala *et al*.^(^ ^5^ ^)^ in determining whether the Mediterranean diet is in fact superior to other dietary interventions.

Another limitation observed in the trials included in the current review was the variations in dietary compliance (see Table 2). The diet that was initially prescribed was not always consistent with what was consumed by the participants. Most studies did, however, manage to create sufficient differences in the consumption of certain macronutrients to allow researchers to distinguish significant differences between the dietary arms. Other researchers, such as Iqbal *et al*.^(^ ^17^ ^)^, reported that there were no significant differences between macronutrient intakes at any point during the trial, and thus it is not surprising that there was no difference in HbA1c levels between the groups.

Even though weight loss was not significantly different between the treatment arms in the included studies, there was a moderate positive correlation between weight loss and HbA1c (data not shown), indicating that higher weight loss was associated with greater improvements in HbA1c. This finding is not surprising, as weight loss is recognised as an integral component of treating patients with T2DM^(^ ^24^ ^)^.

The main strength of this systematic review is that, to our knowledge, it is the first to attempt to control for the effects of weight loss between dietary treatment arms. An additional strength of this review was the use of a recognised tool for assessing the quality of the trials included. A limitation of our review was that, because of the lack of a consistent control diet in the studies examined, we were not able to conduct a meta-analysis or provide quantitative data on the effect of the prescribed diets on changes in HbA1c. It is also not clear whether the participants included in the trials are generally representative of adults with T2DM.

In order to determine whether one particular diet is superior in optimising glycaemic control, a number of research design issues need to be applied in future research studies. First, because of the nature of the effect of weight loss on glycaemic control, it is important to control for this in intervention studies. A well-designed study would include a comparison of dietary interventions that are isoenergetic, and would measure and attempt to balance the energy expenditure of participants. If dietary arms are not isoenergetic, it becomes difficult to distinguish the effects of different macronutrient compositions from the effects of a total energy reduction. Another issue is the need to report medication use and dosage, and ideally control for changes in medication. From the eleven studies included in the current systematic review, only six reported some account of effects of medication. Of these, only Barnard *et al.* ^(^ ^13^ ^)^ and Esposito *et al.* ^(^ ^10^ ^)^ listed the protocols used for how changes in medication were handled. The effects of glucose-lowering medication are clearly of major importance, and if the type and amounts that patients are taking are not controlled for, then the effects of dietary interventions on outcome measures can only be speculative. If more trials address these limitations, it should become clearer whether there is in fact a particular diet that is superior for treating overweight and obese patients with T2DM.

We conclude that there is currently insufficient evidence to state that a particular diet is superior to another for treating overweight and obese adults with T2DM. In line with current ADA guidelines, reducing total energy intake to promote weight loss should be the main strategy. As yet there still is not enough evidence to promote an ideal percentage of energy from carbohydrates, protein and fat. Although the Mediterranean, vegan and low-GI diets appear to be promising, further research that controls for weight loss and the effects of diabetes medications in larger samples is needed.

## Supplementary material

For supplementary material accompanying this paper visit http://dx.doi.org/10.1017/S0007114515003475. click here to view supplementary material
