# Supplementary material for: The effect of macronutrients on glycaemic control: a systematic review of dietary randomised controlled trials in overweight and obese adults with type 2 diabetes in which there was no difference in weight loss between treatment groups
Source: Br J Nutr. 2015 Sep 28;114(10):1656–66. doi: 10.1017/S0007114515003475 (PMC4657029; doi:10.1017/S0007114515003475)
Supplement: Supplementary file 1 [file S0007114515003475sup001.docx]

***Appendix 1. Table of search terms for the 4 databases used***

|  | **Medline** | **Embase** | **CINAHL** | **Web of Science** |
| --- | --- | --- | --- | --- |
| 1 | Diabetes Mellitus, Type 2/ | Diabetes Mellitus, Type 2/ | (MH “Diabetes Mellitus, Type 2”) | diab* near/3 (“type 2” or mellitus) |
| 2 | (diabet* adj3 (“type2” or mellitus)) | (diabet* adj3 (“type2” or mellitus)) | NIDDM | NIDDM |
| 3 | NIDDM | NIDDM | Diabet* n3 (“type 2” or mellitus) | Type 2 diabetes mellitus |
| 4 | Haemoglobin A, Glycosylated/ | Haemoglobin A, Glycosylated/ | (MH “Hemoglobin A, glycosylated”) | Refined by: Diabete mellitus |
| 5 | Haemoglobin a/ or haemoglobin a, glycosylated/ | Haemoglobin a/ or haemoglobin a, glycosylated/ | Glyc* h?emoglobin | Diabetes mellitus |
| 6 | Glyc*h?emoglobin | Glyc*h?emoglobin | HbA1c | 1 or 2 or 3 or 4 or 5 |
| 7 | GHb | GHb | GHb | HbA1c |
| 8 | Glycoh?emoglobin | Glycoh?emoglobin | (MH “Diet+”) | Glyc*h?emoglobin |
| 9 | Diet | Diet | (MH “Diet Therapy+”) | Glycoh?emoglobin |
| 10 | Exp Diet/ | Exp Diet/ | Diet* | GHb |
| 11 | Exp diet therapy/ or diet, reducing/ | Exp diet therapy/ or diet, reducing/ | (calor* or energy*) n2 intak* | Hemoglobin-glycosylated A1c |
| 12 | Diet* | Diet* | (protein* or Mediterranean* or carbohydrate* or fat* or vegetarian* or vegan*) n3 (diet* or intake*) | 7 or 8 or 9 or 10 or 11 |
| 13 | ((calor* or energ*) adj2intak*) | ((calor* or energ*) adj2intak*) | (MH “Dietary Proteins+”) | Diet* |
| 14 | ((protein* or Mediterranean* or carbohydrat* or fat* or vegetarian* or vegan*) adj3 (diet* or intak*)) | ((protein* or Mediterranean* or carbohydrat* or fat* or vegetarian* or vegan*) adj3 (diet* or intak*)) | (MH “Dietary Carbohydrates+”) | (Calor* or energy*) near/2 intak* |
| 15 | Energy intake | Energy intake | (MH “Dietary Fats+”) | (protein* or Mediterranean* or carbohydrate* or fat* or vegetarian* or vegan*) near/3 (diet* or intak*) |
| 16 | Exp energy intake/ | Exp energy intake/ | (MH “Glycemic Index”) OR (MH “Glycemic Load”) | Glyc?emic index |
| 17 | Exp Dietary Proteins/ | Exp Dietary Proteins/ | Glyc?emic index | Glyc?emic load |
| 18 | Exp Dietary Fats/ | Exp Dietary Fats/ | Glyc?emic load | Nutrition* |
| 19 | Exp Dietary Carbohydrates/ | Exp Dietary Carbohydrates/ | Calories | 13 or 14 or 15 or 16 or 17 or 18 |
| 20 | Glycemic Index/ | Glycemic Index/ | Caloric | (19 and 12 and 6) and language: (English) |
| 21 | Glyc?emic index | Glyc?emic index | 1 or 2 or 3 | Randomized controlled trials |
| 22 | Glyc?emic load | Glyc?emic load | 4 or 5 or 6 or 7 | 20 and 21 |
| 23 | Calories | Calories | 8 or 9 or 10 or 11 or 12 or 13 or 14 or 15 or 16 or 17 or 18 or 19 or 20 | Weight* |
| 24 | Caloric | Caloric | 21 and 22 and 23 |  |
| 25 | 1 or 2 or 3 | 1 or 2 or 3 | (MH “Body Weight Changes+”) | 22 and 23 |
| 26 | 4 or 5 or 6 or 7 or 8 | 4 or 5 or 6 or 7 or 8 | Weight * |  |
| 27 | 9 or 10 or 11 or 12 or 13 or 14 or 15 or 16 or 17 or 18 or 19 or 20 or 21 or 22 or 23 or 24 or 25 or 26 or 27 | 9 or 10 or 11 or 12 or 13 or 14 or 15 or 16 or 17 or 18 or 19 or 20 or 21 or 22 or 23 or 24 or 25 or 26 or 27 | 25 or 26 |  |
| 28 | 25 and 26 and 27 | 25 and 26 and 27 | 24 and 27 |  |
| 29 | Limit 28 to (English language and humans and “all dult (19 plus years)” and randomized controlled trial) | Limit 28 to (English language and humans and “all dult (19 plus years)” and randomized controlled trial) |  |  |
| 30 | Body weight / or body weight changes/ or weight gain/ or weight loss/ or overweight/ or thinness/ | Body weight / or body weight changes/ or weight gain/ or weight loss/ or overweight/ or thinness/ |  |  |
| 31 | (weigh* adj3 (los* or gain* or decrease* or increase* or change* or difference*)) | (weigh* adj3 (los* or gain* or decrease* or increase* or change* or difference*)) |  |  |
| 32 | 30 or 31 | 30 or 31 |  |  |
| 33 | 29 and 32 | 29 and 32 |  |  |
